# Spontaneous intracranial hemorrhage in children: report of a hemophilia patient who survived due to a brain cyst

**DOI:** 10.5935/0103-507X.20150069

**Published:** 2015

**Authors:** José Colleti Junior, Walter Koga, Werther Brunow de Carvalho

**Affiliations:** 1 Pediatric Intensive Care Unit, Hospital Santa Catarina - São Paulo (SP), Brazil.; 2 Instituto da Criança, Hospital das Clínicas, Universidade de São Paulo - São Paulo (SP), Brazil.

**Keywords:** Intracranial hemorrhages, Intracranial pressure, Intracranial hypertension/etiology, Hemophilia A/complications, Tomography, x-ray computed, Child, Case reports

## Abstract

We report the case of a 2-year-old child who survived an acute episode of severe spontaneous intracranial hemorrhage with clinical and radiological signs of intracranial hypertension and transtentorial herniation. The patient underwent emergency surgery to drain the hematoma, and a catheter was inserted to monitor intracranial pressure. In the initial computed tomography analysis performed prior to hematoma drainage, a brain cyst was evident contralateral to the hematoma, which, based on the analysis by the care team, possibly helped to avoid a worse outcome because the cyst accommodated the brain after the massive hemorrhage. After the investigation, the patient was determined to have previously undiagnosed hemophilia A. The patient underwent treatment in intensive care, which included the control of intracranial pressure, factor VIII replacement and discharge without signs of neurological impairment.

## Abstract (pt)

Relatamos o caso de um uma criança de 2 anos de idade que sobreviveu após um episódio agudo de hemorragia intracraniana espontânea grave com sinais clínicos e radiológicos de hipertensão intracraniana e herniação transtentorial. O paciente foi para cirurgia de urgência para drenagem do hematoma, sendo inserido um cateter para monitorar a pressão intracraniana. Na análise da tomografia de crânio inicial, antes da drenagem do hematoma, constatou-se um cisto cerebral contralateral ao hematoma que, segundo análise do neurocirurgião e do neuroradiologista, possivelmente evitou um desfecho pior, visto que o cisto serviu de acomodação para o cérebro após a hemorragia maciça. Após investigação, constatou-se tratar de um caso de hemofilia tipo A sem diagnóstico prévio. O paciente foi tratado em terapia intensiva com controle da pressão intracraniana, reposição de fator VIII e obteve alta sem sequelas neurológicas evidentes.

## INTRODUCTION

Severe acute intracranial hemorrhage (ICH) is a life-threatening event associated with high morbidity and mortality.^(1,2)^ It is also associated with an acute increase in intracranial pressure (ICP); as the hematoma increases, the ICP rises, causing non-specific symptoms, such as headache, nausea, vomiting, and changes in the consciousness level. ICH expansion may result in transtentorial herniation, causing neurological deterioration and loss of pupillary reflex.^(3)^ Seizures are common in cases of ICH in pediatric patients.^(4)^

We report the case of a 2-year-old child who presented with an acute episode of severe ICH with intracranial hypertension signs. The cause of ICH was determined to be hemophilia A. In this case, a brain cyst evident on the initial computed tomography scan caught our attention. The care team hypothesized that the cyst was responsible for minimizing the ICP and preventing serious consequences due to transtentorial herniation.

The patient underwent hematoma drainage and ICP monitoring in the intensive care unit (ICU) and received factor VIII administration. The patient was discharged with no obvious neurological sequelae.

## CASE REPORT

A 2-year-old child, 12kg, male, of Japanese descent, with a complaint of drowsiness for one day was evaluated in the emergency room. The patient had no history of previous hospitalizations or comorbidities and had an updated vaccination record. The parents did not report any prevalent disease in the family. In the initial evaluation, the patient was sleepy, pallor +/4, eupneic, afebrile, responding to tactile stimuli with crying, and had a blood pressure of 100 x 40mmHg and 130mg% blood glucose levels. Volume replacement was prescribed at 20mL/kg with 0.9% saline, and laboratory tests were performed upon admission. The initial diagnosis was exogenous intoxication, although the parents denied any possibility of such.

**Figure 1:**
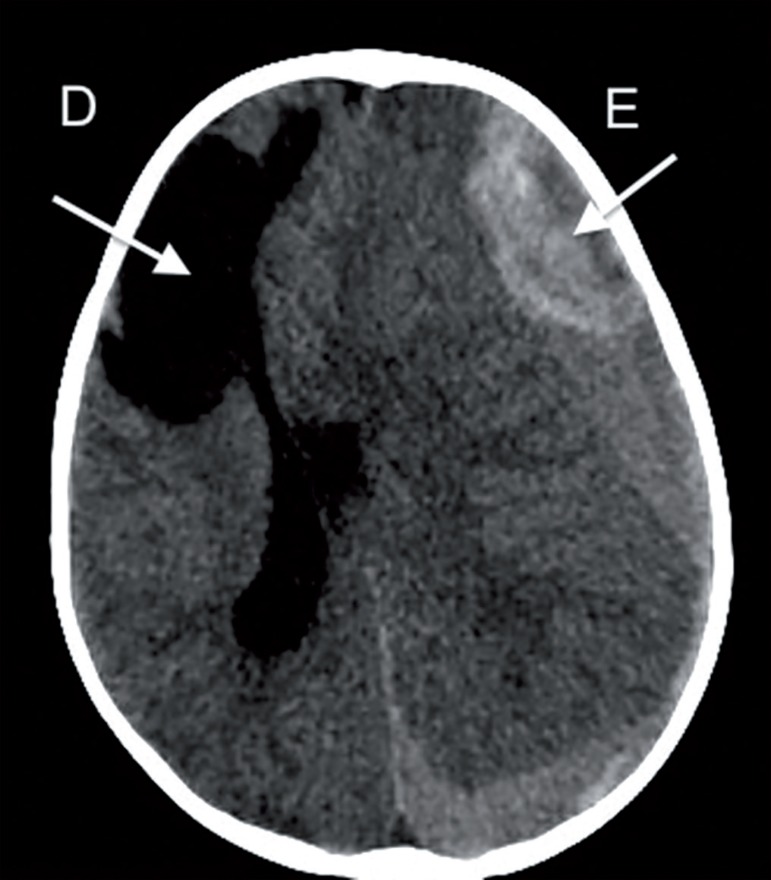
Initial computed tomography scan of the skull: evidence of a brain cyst on the right and intracranial hemorrhage on the left (arrows), with signs of intracranial hemorrhage and midline shift.

While under clinical observation in the emergency department, the patient presented with a generalized tonic-clonic seizure with lip cyanosis and a decrease in oxygen saturation to 94%, lasting for approximately 1 minute. The treating physician described the patient as unresponsive to verbal stimuli, without spontaneous eye opening, mydriatic left pupil not photoresponsive, isochoric right pupil with ipsilateral and contralateral photoreaction, without meningeal signs. Emergency computed tomography of the skull was performed (Figure 1), where subdural hemorrhage was evident, bypassing the left brain hemisphere. This hemorrhage was heterogeneous, with apparent active bleeding, exhibiting an estimated maximum thickness of approximately 2.7cm in the frontal region, exerting remarkable compression on the neighboring brain parenchyma, promoting a midline shift to the right by approximately 1.9cm at the level of the septum pellucidum with signs of subfalcine herniation of the cingulate gyrus, and transtentorial descending and lateral (uncal and parahippocampal) herniation, significantly compressing the midbrain. The dimensions of the second and fourth ventricles were significantly reduced in size due to the compressive effect of the brain herniation. The computed tomography also revealed a cystic lesion, apparently a sequela, compromising the right frontal lobe.

Meanwhile, the patient's neurological condition deteriorated, and the Glasgow Coma Scale score decreased to 6. The patient was intubated using rapid sequence intubation, and mechanical ventilation was started. The neurosurgeon was alerted, who proceeded to urgently drain the hematoma and install the ICP catheter. The diagnostic tests upon admission revealed a prothrombin time of 13.4 seconds with an international normalized ratio (INR) of 0.97 and an activated partial thromboplastin time (aPTT) of 94 seconds (patient/normal [P/N]: ratio 2.63). Other tests were within normal limits. The mother was asked about previous bleeding and reported an occasion where, after the collection of routine tests, the child presented with a significant hematoma at the puncture site. At the time, he was advised to seek medical attention but did not.

The patient was transferred to the pediatric ICU and remained on mechanical ventilation, pressure controlled mode, using capnography, targeting carbon dioxide tension at the end of expiration (EtCO_2_) between 35 and 45mmHg while also avoiding hyperoxia. He received sedoanalgesia with thiopental (60mcg/kg/min), fentanyl (4mcg/kg/min) and midazolam (0.4mcg/kg/min). He also received hidantal at a loading dose of 15mg/kg and a maintenance dose of 5mg/kg/day. He continued to receive noradrenaline (0.2mcg/kg/min) to maintain a cerebral perfusion pressure between 40 and 65mmHg. A central catheter was peripherally inserted in the right basilar vein, and a 4F double-lumen central venous catheter was inserted in the right internal jugular vein. The patient remained fasting, receiving isotonic maintenance fluids with 100mL/kcal and basal electrolytes on the first day; on the second day, total parenteral nutrition was started. The ICP was monitored to maintain it below 20mmHg.

The tests were repeated and the results are as follows: INR at 0.99; aPTT at 80 seconds with a P/N ratio of 2.37; and fibrinogen at 328mg/dL (normal: 200 - 400mg/dL). The patient was evaluated by the pediatric hematology team, and factor VIII (23%; normal: 57% - 192%) and factor VIII (undetectable) inhibitors were measured, revealing a clinical picture of hemophilia A. We began immediate intravenous administration of factor VIII.

On the third day of hospitalization, we started to reduce thiopental aided by continuous electroencephalography.

**Figure 2:**
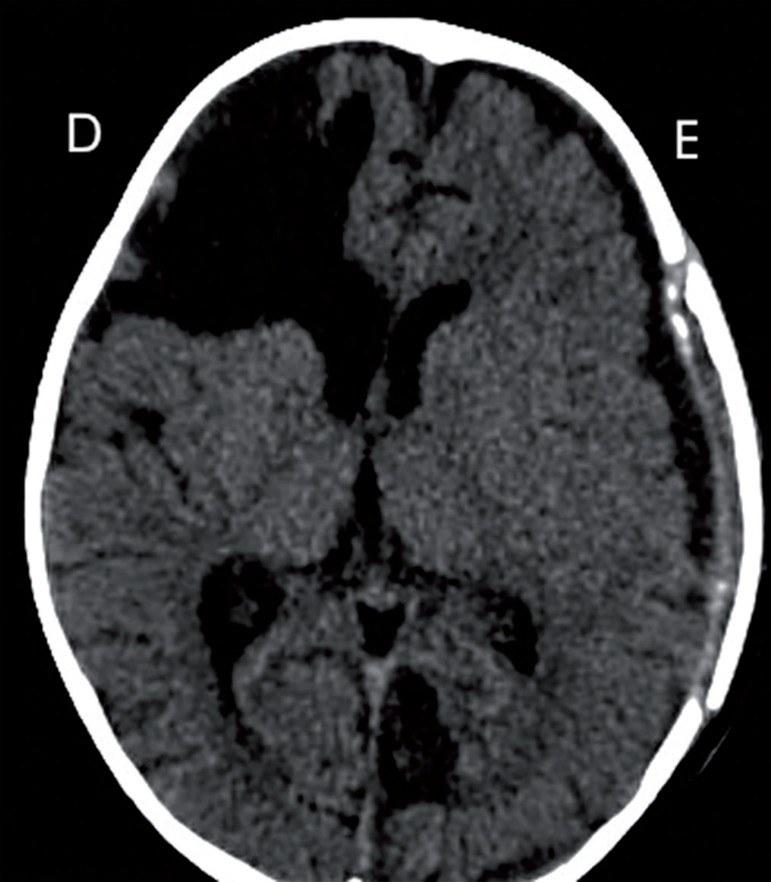
Computed tomography scan of the skull prior to discharge showing brain remodeling.

The patient was successfully extubated on the 19^th^ day of hospitalization. He developed ptosis of the left eyelid. He gradually recovered motor and intellectual abilities and spoke normally, and the ptosis decreased. In the control computed tomography of the skull (Figure 2), note the new brain architecture with re-accommodation of the brain parenchyma without signs of hematomas. Currently, the patient is being monitored at a specialized clinic for hemophilia patients.

## DISCUSSION

Spontaneous ICH is associated with a high mortality and morbidity.^(5)^ The incidence is estimated at 24.6 cases per 100,000 people annually, with mortality rates of 40% at 1 month and 54% at 1 year, with only 12% to 39% of patients recovering long-term functional independence.^(5)^ Spontaneous ICH is responsible for 50% of strokes in children, whereas it accounts for only 15% of strokes in adults. In adults, hypertension is the most common cause of ICH, whereas in children, secondary factors such as vascular malformations cause ICH, although there are still few pediatric studies.^(6)^ ICH is an important cause of morbidity and mortality in hemophilia patients, with an incidence ranging from 2.2 to 7.5%.^(7)^ Clotting factor replacement therapy, emergency neurosurgery and rapid and appropriate airway management are essential in comatose patients.^(7)^ A multidisciplinary approach involving hematologists, neurosurgery and intensive care personnel is crucial for achieving a favorable outcome. The decrease in 30-day mortality is likely related to the introduction of investigation protocols, early diagnosis and management strategies for these patients, including monitoring in ICU environments.^(8,9)^

In our case report, the patient was already presenting with signs of transtentorial herniation on the initial computed tomography of the skull, given the large extent of the hemorrhaging. Signs of neurological deterioration were rapid, following increasingly severe degrees of drowsiness, seizures and unilateral mydriasis. What was notable here and the consensus among the various teams who assisted the patient was that the brain cyst accommodated the pressure and prevented a more tragic outcome for the patient. There are reports of patients with congenital blood dyscrasias who have presented with brain cysts due to previous bleeding events that have been reabsorbed.^(10)^ We suspect that this patient's brain cyst may have originated from a prior bleeding event, possibly even during the intrauterine period.

## CONCLUSION

This report emphasizes the importance of the early recognition of signs of intracranial hemorrhage in children, the pursuit of the underlying cause and immediate treatment provided by the neurosurgical team, in addition to monitoring in the intensive care unit using intracranial hemorrhage management protocols. Sustained intracranial hemorrhage and cerebral herniation are neurological emergencies. Similarly to cardiac arrest, a neurologic emergency also demands an organized management algorithm for the care of critically ill patients. The goal of having a neurologic emergency protocol is to establish standardized evidence-based management for patients with intracranial hemorrhage and/or transtentorial herniation.

Thus, even with extensive intracranial hemorrhaging, the patient not only survived a catastrophic event but also adequately regained his neurological functions. In this case report, we would like to highlight computed tomography images of the skull, in which a brain cyst seemed to accommodate the pressure caused by the hematoma, helping to avoid a more dramatic outcome.
